# Enhanced external counterpulsation improves cardiac function in Beagles after cardiopulmonary resuscitation

**DOI:** 10.1590/1414-431X20199136

**Published:** 2020-01-13

**Authors:** Jing Xiong, Wei Zhang, Hongyan Wei, Xin Li, Gang Dai, Chunlin Hu

**Affiliations:** 1 Cadre's Ward, The First Affiliated Hospital of Nanchang University, Nanchang, Jiangxi, China; 2 Respiratory Department, The First Affiliated Hospital of Nanchang University, Nanchang, Jiangxi, China; 3 Department of Emergency Medicine, The First Affiliated Hospital, Sun Yat-sen University, Guangzhou, Guangdong, China; 4 Department of Emergency, Guangdong Provincial People's Hospital, Guangzhou, Guangdong, China; 5 NHC Key Laboratory of Assisted Circulation, Sun Yat-sen University, Guangzhou, Guangdong, China

**Keywords:** Cardiopulmonary resuscitation, Hemodynamics, Enhanced external counterpulsation, PiCCO2, Myocardial blood flow, Beagle dog

## Abstract

The aim of this study was to investigate the influence of enhanced external counterpulsation (EECP) on the cardiac function of beagle dogs after prolonged ventricular fibrillation. Twenty-four adult male beagles were randomly divided into control and EECP groups. Ventricular fibrillation was induced in the animals for 12 min, followed by 2 min of cardiopulmonary resuscitation. They then received EECP therapy for 4 h (EECP group) or not (control group). The hemodynamics was monitored using the PiCCO2 system. Blood gas and hemorheology were assessed at baseline and at 1, 2, and 4 h after return of spontaneous circulation (ROSC). The myocardial blood flow (MBF) was quantified by ^18^F-flurpiridaz PET myocardial perfusion imaging at baseline and 4 h after ROSC. Survival time of the animals was recorded within 24 h. Ventricular fibrillation was successfully induced in all animals, and they achieved ROSC after cardiopulmonary resuscitation. Survival time of the control group was shorter than that of the EECP group [median of 8 h (min 8 h, max 21 h) *vs* median of 24 h (min 16 h, max 24 h) (Kaplan Meyer plot analysis, P=0.0152). EECP improved blood gas analysis findings and increased the coronary perfusion pressure and MBF value. EECP also improved the cardiac function of Beagles after ROSC in multiple aspects, significantly increased blood flow velocity, and decreased plasma viscosity, erythrocyte aggregation index, and hematocrit levels. EECP improved the hemodynamics of beagle dogs and increased MBF, subsequently improving cardiac function and ultimately improving the survival time of animals after ROSC.

## Introduction

In the United States, approximately 300,000 people have an out-of-hospital cardiac arrest each year, and approximately 30–40% of those cases achieve return of spontaneous circulation (ROSC), with only 7% of patients surviving until hospital discharge (1,2). Most patients die from post-cardiac arrest syndrome, which is a complex constellation of multi-organ failure, comprising brain injury, systemic ischemia-reperfusion response, and post-cardiac arrest myocardial dysfunction. (2,3). Myocardial dysfunction is an important cause of post-resuscitation circulatory failure that may lead to early mortality after ROSC (3,4). It is defined as a reversible global dysfunction due to a stunned myocardium in the absence of coronary occlusion and microcirculation disorder of the coronary artery (5–9).

Hemodynamics is very complicated after ROSC, and although a stable circulation may be indicated, the ischemia, hypoxia, and metabolic state of tissues may not truly be represented. Clinical and experimental studies have found that, in some situations, persistent tissue hypoxia may lead to organ damage despite the restoration of global hemodynamic parameters (heart rate, mean artery pressure, and cardiac index) to their “normal” levels (10–12). Clinical interventions to improve microcirculatory blood flow are still very limited.

Enhanced external counterpulsation (EECP), which was introduced in the American Heart Association/American College of Cardiology Guideline of Coronary Artery Disease in 2002, has been proven to be an effective, safe, and economical therapy for the management of ischemic cardiovascular and cerebrovascular diseases (13). The device for EECP consists of inflatable cuffs that encircle the calf, thigh, and upper thigh. The cuffs squeeze sequentially from low to high during diastole and then rapidly, and simultaneously, deflate at the onset of systole (14). The mechanism regulating the device is based on an electrocardiogram. The arterial hemodynamics that are generated by the device simulate those of the intra-aortic balloon pump with the generation of a retrograde arterial wave pulse. However, unlike an intra-aortic balloon pump, a retrograde venous pulse is also generated, which increases venous return. The retrograde arterial wave pulse causes augmented coronary flow, whereas the improved venous return helps to improve cardiac output and decrease oxygen consumption (VO_2_) (15). The systolic deflation/diastolic inflation sequence of EECP leads to systolic unloading and diastolic augmentation, resulting in a pulsatile increase in blood flow. EECP has been proven to be able to augment shear stress of blood flow, and thus improve the function of vascular endothelial cells, which in turn improves microcirculation (16). In a recent study, we found that EECP treatment after ROSC improved neurological function in beagle dogs, increased the release of vascular endothelial-derived relaxing factor NO-1, and increased brain microcirculation blood flow (14). However, whether EECP can improve myocardial blood flow (MBF) after cardiopulmonary resuscitation (CPR) is still unclear.

The PiCCO2 is a device for continuous cardiac output measurement combined with monitoring of cardiac preload volume, extravascular lung water (EVLW), and arterial and central venous oxygen saturation (ScvO_2_). The PULSION PiCCO2 computes the cardiac output continuously, utilizing an improved arterial pulse contour analysis algorithm. The pulse contour cardiac output (PCCO) is calibrated by means of a transpulmonary thermodilution measurement. A cold or room-temperate bolus (e.g., normal saline 0.9%) is injected through a central venous catheter. A thermodilution curve is recorded by an arterial thermodilution catheter, which is also used for pressure monitoring. In addition to PCCO calibration, transpulmonary thermodilution also yields cardiac preload by means of global end-diastolic volume (GEDV) and estimation of both intrathoracic blood volume (ITBV) and EVLW. Furthermore, the PiCCO2 continuously measures ScvO_2_ after calibration with blood gas analysis results and can continuously calculate oxygen delivery (DO_2_) and VO_2_ (17–19). Therefore, in addition to monitoring hemodynamics, PiCCO2 can also reflect tissue perfusion and is an ideal monitoring tool after ROSC.

The effects of EECP on hemodynamics are complex, and the PiCCO2 system is a comprehensive tool for assessing hemodynamics. For the first time, we used the PiCCO2 system to monitor the effects of EECP on the systemic hemodynamics of dogs after ROSC. We aimed to investigate the influence of EECP on systemic hemodynamics, MBF, and tissue oxygenation of beagle dogs after ROSC.

## Material and Methods

### Experimental protocol

This study was performed on 24 healthy male adult beagle dogs (body weight 12.6–15 kg) and was approved by the Institutional Animal Care and Use Committee, Sun Yat-sen University (2013A-067). Animals were managed according to the guidelines of the American Physiological Society. The dogs were randomly divided into control and EECP groups, with 12 animals in each group. Weight was accurately measured using a scale, and temperature inside the anus was assessed using a probe thermometer. Original respiratory rate was manually counted, and heart rate and femoral blood pressure were derived from results of ECG and blood pressure monitoring. Before surgery, all animals fasted overnight, but had free access to water.

Animals were anesthetized with intraperitoneal injection of 30 mg/kg phenobarbital and, if necessary, a 1/4 dose was administered to maintain anesthesia effects. Dogs were placed in the supine position, and a 7.5-mm endotracheal tube (Becton Dickinson Medical Devices Co., Ltd., China) was inserted during spontaneous respiration. Ventilation was performed using a Servo ventilator (Servo 900, Siemens, Germany) with room air at 20 breaths/min. Ventilation parameters were adjusted using arterial blood gas results to maintain PCO_2_ levels in the range of 35–45 mm Hg. A flow chart is shown in Supplementary Figure S1.

### Hemodynamic measurements

Dogs were placed in supine position, with limbs fixed. The right anterior tibialis was dissected, and a 6-F arterial catheter was inserted to measure the blood pressure and cardiac output by means of a femoral arterial thermodilution system (PiCCO2, Pulsion Medical Systems, Germany). Continuous hemodynamic surveillance was initiated using arterial pulse contour analyses with transpulmonary thermodilution calibration. At least three boluses of iced (<8°C) 0.9% saline were injected through the central venous catheter into the right internal jugular vein, and the thermodilution curve was used to estimate hemodynamic variables. Three consecutive injections for calibration were performed at set up, repeated at each hour, or more frequently if indicated by changes in condition.

The left common carotid artery was punctured, and a 6-F arterial pressure catheter was placed into the ascending aorta root. The transducer was connected to the MP150 analysis BIOPAC system to record the direct arterial pressure. The right internal jugular vein puncture was placed into the venous catheter to the right atrium (or inferior vena cava) and connected to a central venous pressure tube to measure central venous pressure. Coronary perfusion pressure (CPP) was calculated by subtracting the mid-diastolic right atrial pressure from the mid-diastolic aortic pressure. A standard lead II electrocardiogram was used to monitor cardiac rhythm (MP150). An intravenous indwelling needle was inserted into the right ear vein for administration and supplementation of physiological energy requirements of the animal.

### Induction of ventricular fibrillation

Referring to our previous research methods (14), ventricular fibrillation was induced by alternating current (50 V, 50 Hz) stimulation of the external epicardium. Cardiac arrest judgment criteria were as follows (20): 1) systolic blood pressure rapidly drops <25 mmHg after electrical stimulation; 2) arterial pulsation waveform disappears; and 3) ECG waveform indicates ventricular fibrillation. The duration of cardiac arrest was calculated from the time the blood pressure dropped <25 mmHg after electrical stimulation. All data were recorded in the Utstein style.

### Cardiopulmonary resuscitation

After 12 min of ventricular fibrillation, CPR was performed according to the US 2015 Cardiopulmonary Resuscitation Guideline (21). An external defibrillating countershock (SAN-ei Cardiopace 3 MII, USA) of 5 J/kg was delivered and repeated, as needed, in rapid sequence. Immediately afterwards, CPR was performed, and 20 μg/kg adrenaline was administered at 3-min intervals. Chest compression frequency was 100 times/min, compression depth was 4–5 cm (front and back breast diameter), and compression relaxation ratio was 1:1. Ventilator (PA-500) controlled ventilation had a frequency of 10 times/min, tidal volume of 10 mL/kg, and inhaled oxygen concentration of 40%. During the experiment, ventilator parameters were adjusted according to blood gas analysis results, and the PCO_2_ was controlled between 35 and 45 mmHg. ROSC criteria were to restore supraventricular rhythm, mean arterial pressure ≥60 mmHg, and maintain this for >10 min (20). The survival status of the animals within 24 h after ROSC was recorded. Animals that did not respond or had absence of breath and pulse were considered dead.

### EECP protocol

After ROSC, animals in the EECP group immediately received EECP treatment (Shuangshan EECP-MCI, China) for 4 h. EECP was carried out according to our previous methods (14). Briefly, EECP was performed after laying the dogs on their left side and wrapping two sets of cuffs that were modified to closely fit canine lower extremities and hips. Using electrocardiogram gating, the cuffs were sequentially inflated with compressed air from distal to proximal in early diastole and were rapidly deflated immediately before systole. The pressure applied to the cuffs was set at 0.040 mPa/cm^2^. EECP procedures were also performed on animals in the control group, but the cuffs were not sequentially inflated.

### Blood gas analysis and blood lactate detection

Arterial blood was analyzed (i-STAT System 300, ABBOTT, USA) for pH, PCO_2_, base excess, PO_2_, HCO_3_-, SO_2_, and lactic acid at baseline and after ROSC for 4 h.

### Hemorheology test

Whole blood (4 mL) was extracted into a heparin anticoagulant tube at baseline and 1, 2, and 4 h after ROSC to detect whole blood viscosity, hematocrit, erythrocyte aggregation index, and blood flow velocity (LBY.N6A, China).

### ^18^F-flurpiridaz PET MPI

Positron emission tomography (PET) myocardial perfusion imaging (MPI) scanning was performed in the anesthetized dogs in the supine position on a movable table top, with the head fixed inside the scanner (Philips Gemini GXL 16, Philips Medical System (USA) Inc., Netherlands). The PET scanner has sixteen detector rings, generating four main slices and three cross-slices, with a thickness of approximately 2 mm. Sensitivity was 675 cps/kBq/mL for the main slices and 945 cps/kBq/mL for the cross-slices, and the spatial resolution was 8 mm. The axial range was 81 mm. We used an image matrix of 512×512 pixels with a pixel size of 1.44×1.44 mm^2^. All animals had been fasted for at least 8 h before starting PET/computed tomography (CT) examination and remained under anesthesia throughout the entire experiment. ^18^F-flurpiridaz was administered by hand as a bolus injection into the femoral vein and flushed with 5-mL saline solution (injection time of 5 s). The protocol was repeated at baseline (before cardiac arrest), and 4 h after ROSC. Results were quantitatively analyzed using Myovation (GE Healthcare, USA) and QGS/QPS software (Cedars Quantitative Perfusion SPECT, Version 4.0, Cedars-Sinai Medical Center, USA).

### Statistical analyses

All data were analyzed by SPSS 13.0 software (USA) and are reported as means±SD or quartiles according to the results of the normal distribution test. Comparison between two sample means was performed using the independent *t*-test or Mann-Whitney test. Comparison between multiple samples was done using one-way analysis of variance (ANOVA) and *post-hoc* multiple comparisons test or Kruskal-Wallis H. The Kaplan Meyer plot analysis was used to compare the survival rate between two groups. P<0.05 was statistically significant.

## Results

### Physiological and CPR-related parameters

Respiratory rate was 16±1 breaths/min, heart rate was 133±8 beats/min, and body temperature (anal temperature) was 38.15±0.49°C. Ventricular fibrillation was successfully induced in all animals, and they obtained ROSC after CPR. There was no significant difference in the basic physiological and CPR-related parameters between the two groups of animals. The specific values are shown in Supplementary Table S1. Three animals (3/12) in the control group and 7 animals in the EECP group survived for 24 h. The survival time of the control group was a median of 8 h (min 8 h, max 21 h), which was significantly shorter than the median of 24 h (min 16 h, max 24 h) of the EECP group (P=0.026). Kaplan Meyer plot analysis showed that the fraction survival of animals in the EECP group was higher than that in the Control group (P=0.0005). Blood gas analysis results showed that after ROSC, the control group showed serious metabolic acidosis, demonstrated by decreased HCO_3_- concentrations, decreased base excess, and increased blood lactate levels, whereas the metabolic acidosis of the EECP group was significantly milder than that of the control group (Table 1).

**Table 1:** Blood gas parameters at baseline and at 2 and 4 h after return of spontaneous circulation in Control and enhanced external counterpulsation (EECP) groups.

Group	Basal	2 h	4 h
pH			
Control	7.4±0.1	7.3±0.3	7.3±0.3
EECP	7.4±0.2	7.3±0.3	7.4±0.2**
PCO_2_ (mmHg)			
Control	42.8±3.6	45.5±1.1	39.4±4.3
EECP	43.4±3.7	41.3±6.0	38.1±1.8
PO_2_ (mmHg)			
Control	84.0±12.3	82.0±1.7	82.3±3.1
EECP	82.7±6.7	81.7±5.7	85.7±2.5
BE			
Control	-2.0±1.0	-6.7±0.6	-6.3±0.6
EECP	-2.0±1.0	-3.3±0.6***	-3.0±1.0***
HCO_3_- (mmol/L)			
Control	22.8±0.1	18.1±1.1	19.8±0.2
EECP	23.1±0.7	23.7±0.3***	23.1±0.7***
SO_2_ (%)			
Control	98.0±1.7	96.3±2.1	96.7±1.2
EECP	97.3±2.1	97.7±2.3	95.7±0.6
FIO_2_ (%)			
Control	21.0±0.0	21.0±0.0	21.0±0.0
EECP	21.0±0.0	21.0±0.0	21.0±0.0
LAC (mmol/L)			
Control	1.4±0.1	5.0±1.2	4.0±0.3
EECP	1.5±0.2	2.8±0.2***	2.5±0.7***

pH: power of hydrogen; PCO_2_: partial pressure of carbon dioxide; PO_2_: partial pressure of oxygen; BE: base excess; HCO_3_-, bicarbonate; SaO_2_: arterial oxygen saturation; FIO_2_: fraction of inspired O_2_; LAC: lactic acid. Data are reported as means±SD. **P<0.01, ***P<0.001 between groups (*t*-test).

### EECP improved hemodynamics after ROSC

There was no significant difference in heart rate between the two groups at each time point after ROSC. Mean artery pressure was significantly higher in the EECP group than in the control group. Central venous pressure was slightly higher in the EECP group, but the difference was not significant. However, CPP was significantly higher in the EECP group (Table 2).

**Table 2:** Comparison of hemodynamic parameters between Control and enhanced external counterpulsation (EECP) groups after return of spontaneous circulation (ROSC).

	Baseline	ROSC 1 h	ROSC 2 h	ROSC 3 h	ROSC 4 h
HR (times/min)					
Control	196.4±3.4	168.7±16.4	161.9±8.5	167.3±2.8	167.3±3.3
EECP	204.5±12.9	167.5±6.2	162.5±1.3	165.0±1.7	169.9±2.0
APm (mmHg)					
Control	153.7±3.6	109.6±56.6	94.7±10.6	114.9±8.2	123.6±5.1
EECP	151.5±3.9	108.4±40.4	125.6±1.9***	139.3±6.1***	148.2±2.3***
CVP (mmHg)					
Control	8.0±1.2	8.4±2.3	9.2±2.3	9.4±1.5	9.8±1.9
EECP	7.6±1.9	8.8±2.6	9.8±3.1	9.5±2.4	10.2±2.1
CPP (mmHg)					
Control	143±13	99±16	84±12	92±12	110±15
EECP	141±14	101±20	108.6±19***	119±16***	136±13***

HR: heart rate; APm: mean artery pressure, CVP: central venous pressure; CPP: coronary perfusion pressure. Data are reported as means±SD. ***P<0.001 EECP group *vs* Control group (*t*-test).

In the EECP group, the myocardial contractility of dogs was decreased after ROSC and the values of maximum rate of increase in pressure, CO, global ejection fraction, and PCCO were decreased, but the cardiac function of the animals was improved. The values of these parameters were significantly higher in the EECP group than in the control group. GEDV and ITBV of the EECP group after ROSC were significantly higher, indicating that EECP promoted blood return to the chest and heart. Systemic vascular resistance was significantly lower in the EECP group, indicating that EECP can reduce peripheral vascular resistance and increase stroke volume. EVLW and pulmonary vascular permeability index (PVPI) of the two groups were increased at each time-point after ROSC, indicating that there may be a lung injury after ROSC, but the EVLW and PVPI were significantly lower in the EECP group. ScvO_2_ and DO_2_ values at each time-point after ROSC were significantly higher in the EECP group, whereas the VO_2_ values were the opposite, indicating that EECP can increase DO_2_ and reduce tissue VO_2_. This result is consistent with the previous EECP results of reducing blood lactate levels after ROSC (Table 3).

**Table 3:** Comparison of PiCCO2 parameters between Control and enhanced external counterpulsation (EECP) groups after return of spontaneous circulation (ROSC).

Group	Baseline	ROSC 1 h	ROSC 2 h	ROSC 3 h	ROSC 4 h
PCCO (L/min)					
Control	3.4±0.2	2.0±0.7	1.2±0.1	1.9±0.3	1.8±0.1
EECP	3.3±0.3	2.1±0.5	2.8±0.3***	2.4±0.3**	2.3±0.2**
SV (mL)					
Control	17.9±0.9	12.6±4.3	12.2±2.9	11.9±2.1	8.1±0.6
EECP	18.0±0.3	17.6±3.6***	17.2±1.7***	14.6±1.7***	13.4±1.1***
SVR (dyn*s*cm^-5^)					
Control	4209.2±164.8	4080.2±2186.8	10597.8±7579.6	7714.1±1032.5	7238.6±558.8
EECP	4165.9±205.4	3881.0±514.2**	3498.8±422.5***	4593.9±669.9***	5095.7±517.2***
DPMX (mmHg/s)					
Control	1489.4±190.7	933.0±58.3	585.5±38.1	686.2±57.5	717.0±43.8
EECP	1483.0±274.6	951.3±48.2	993.3±65.1***	1036.1±96.3***	1128.5±51.3***
CO (L/m)					
Control	3.5±0.4	2.1±0.5	1.4±0.2	1.4±0.3	1.8±0.5
EECP	3.4±0.6	2.8±0.3*	2.0±0.6***	1.7±0.2***	2.8±0.7***
GEDV (mL)					
Control	198.0±15.3	143.0±9.0	140.0±25.8	181.1±16.9	188.0±19.9
EECP	198.5±13.1	198.0±0.0***	219.4±25.6***	226.3±19.4**	238.2±33.2***
ITBV (mL)					
Control	247.6±3.9	247.0±0.0	273.7±19.7	272.6±61.6	235.1±41.2
EECP	250.0±9.8	278.8±11.2**	295.0±18.6**	296.4±51.2***	285.0±61.8***
EVLW (mL)					
Control	136.7±1.2	145.5±54.3	346.5±44.9	342.4±28.3	248.6±46.5
EECP	135.0±3.8	153.6±10.3*	157.0±32.4***	159.6±21.1***	160.0±39.1***
PVPI					
Control	2.7±0.8	3.7±0.7	6.5±2.3	6.3±1.1	6.3±1.4
EECP	2.9±0.5	3.3±0.5*	4.5±1.2**	3.5±0.4***	3.3±0.9***
GEF (%)					
Control	35.0±0.2	35.2±1.1	20.5±3.7	20.4±1.5	20.3±2.6
EECP	34.0±0.5	36.6±1.3	37.0±5.2***	29.9±7.1**	27.0±1.7***
ScvO_2_ (%)					
Control	80.1±2.4	60.3±10.6	62.5±6.1	67.6±7.5	70.9±6.4
EECP	80.1±1.8	80.0±4.1***	85.1±0.9***	82.7±1.5***	75.1±2.8**
DO_2_ (mL/min^.^m^2^)					
Control	731.6±41.1	658.6±154.4	594.7±98.6	408.4±76.2	279.5±23.8
EECP	725.8±44.1	702.4±119.7	669.4±64.9**	569.2±65.2**	542.3±45.2***
VO_2_ (mL/min^.^m^2^)					
Control	101.5±19.4	321.7±104.9	289.4±50.6	272.2±9.7	161.7±25.0
EECP	104.6±12.3	280.8±39.7**	269.6±8.5*	249.8±61.5**	114.2±19.3***

PCCO: pulse contour cardiac output; SV: stroke volume; SVR: systemic vascular resistance; DPMX: maximum rate of increase in pressure; CO: cardiac output; GEDV: global end-diastolic volume; ITBV: intrathoracic blood volume; EVLW: extravascular lung water; PVPI: pulmonary vascular permeable index; GEF: global ejection fraction; ScvO_2_: central venous oxygen saturation; DO_2_: oxygen delivery; VO_2_: oxygen consumption. Data are reported as means±SD. **P<0.05, **P<0.01, ***P<0.001 EECP group *vs* Control group (*t*-test).

### EECP improved the hemorheology of dogs after ROSC

**Figure 1:**
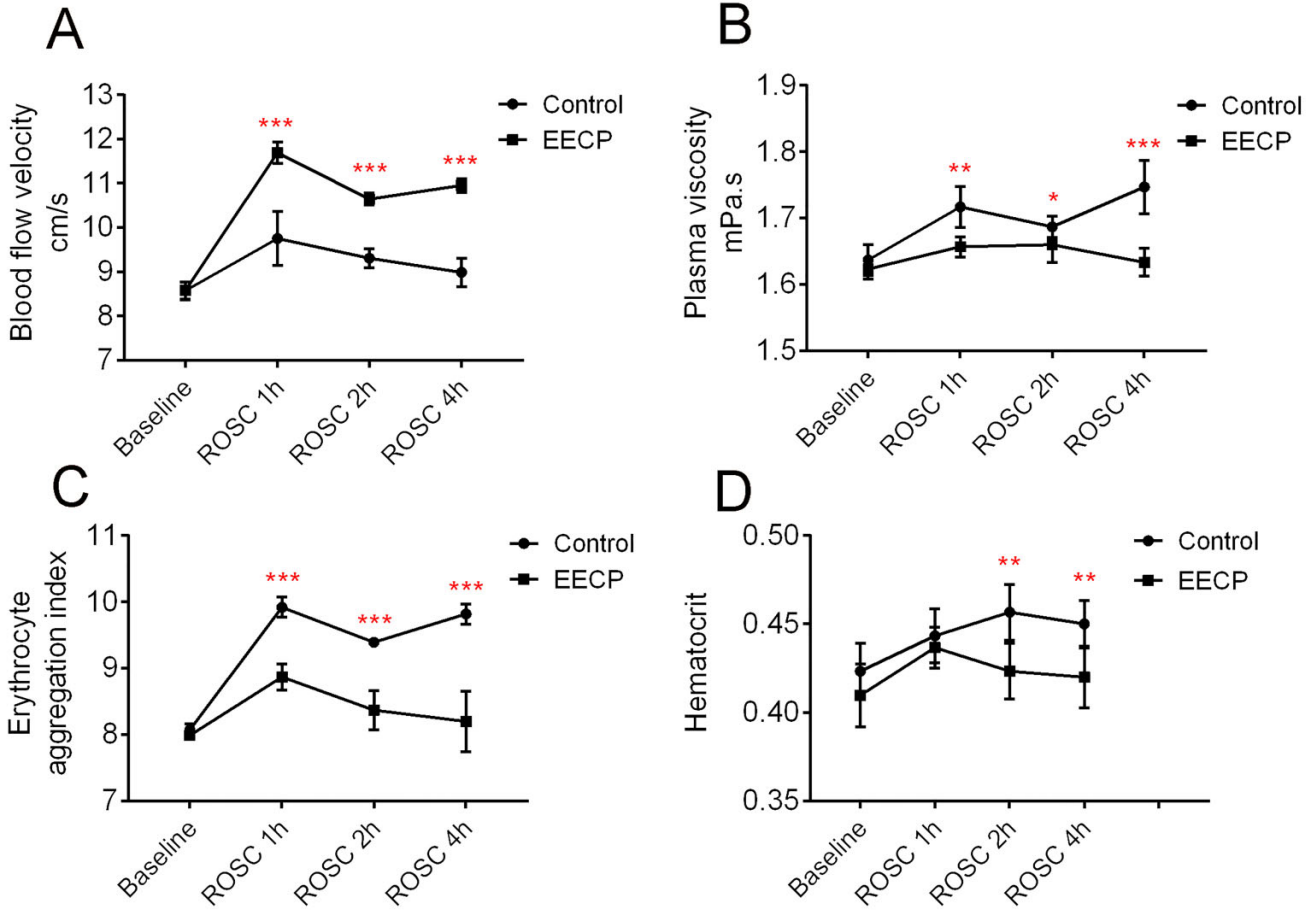
Enhanced external counterpulsation (EECP) improved the hemorheology of dogs after return of spontaneous circulation (ROSC). **A**, EECP increased blood flow velocity; **B**, EECP decreased plasma viscosity; **C**, EECP decreased erythrocyte aggregation index; **D,** EECP slightly decreased hematocrit. Data are reported as means±SD. *P<0.05, **P<0.01, ***P<0.001 (ANOVA).

Compared with the control group, the EECP group after ROSC had significantly increased blood flow velocity (Figure 1A), and decreased plasma viscosity (Figure 1B), erythrocyte aggregation index (Figure 1C), and hematocrit (Figure 1D). Hemorheology parameters were affected by temperature, blood glucose concentration, and hemoglobin level, but there was no significant difference between the two groups after ROSC (Supplementary Table S2).

### EECP improved myocardial blood flow

**Figure 2:**
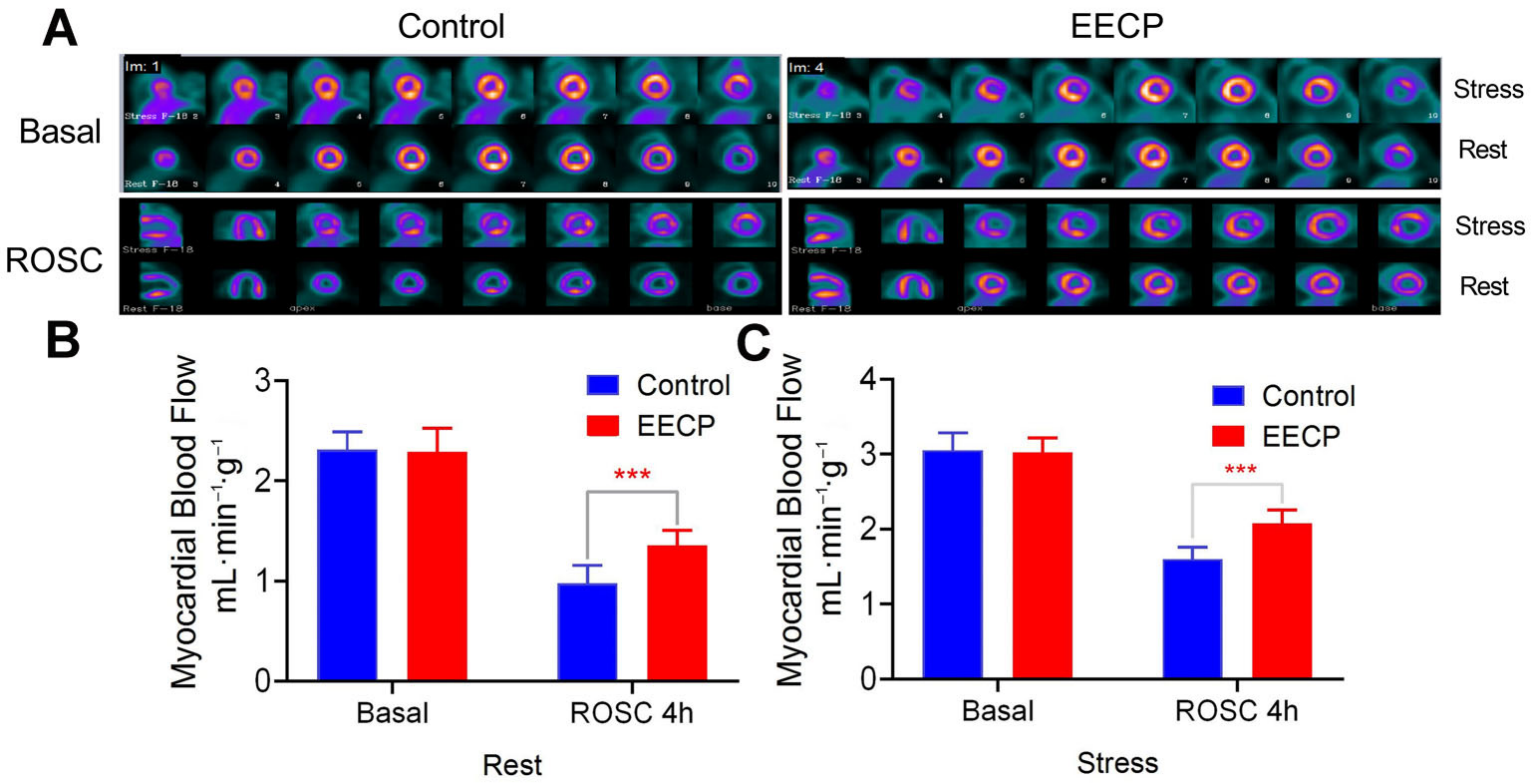
**A**, Enhanced external counterpulsation (EECP) improved the myocardial blood flow (MBF) after return of spontaneous circulation. The MBF of the EECP treatment group was significantly higher than that of the control group, both at rest (**B**) and under dobutamine stress condition (**C**). Data are reported as means±SD. ***P<0.001 (*t*-test).

**Figure 3:**
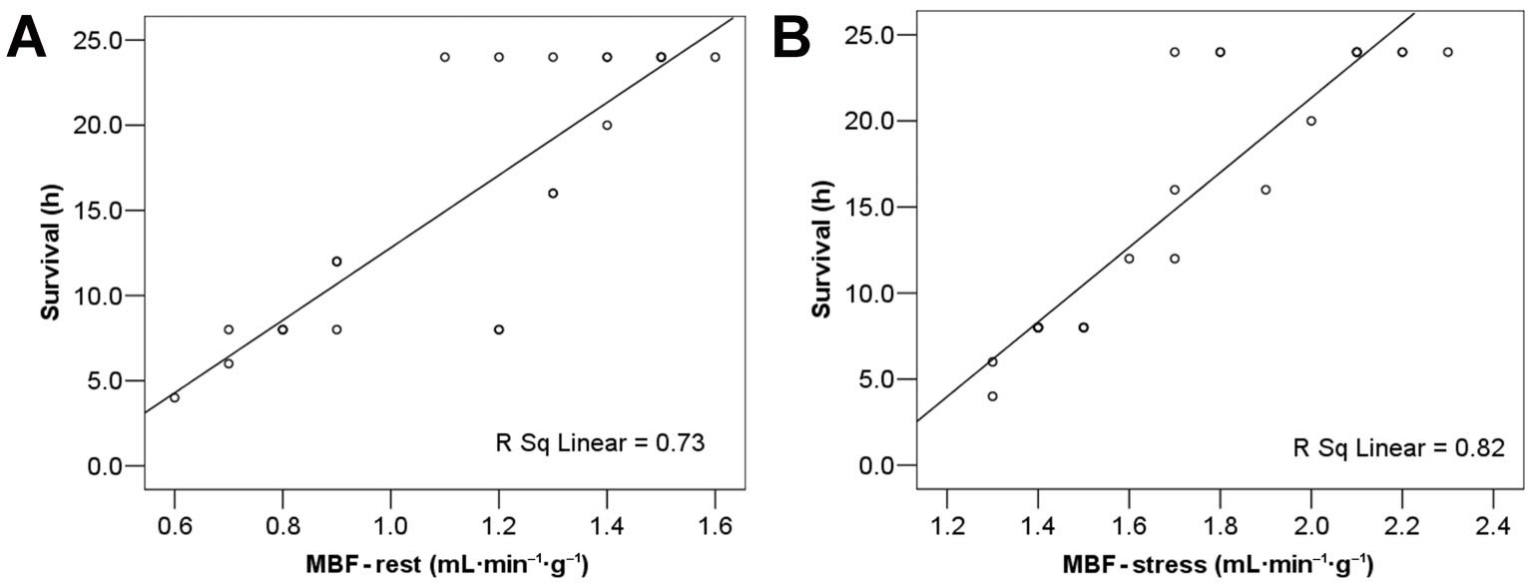
Correlation between myocardial blood flow (MBF) and survival time of dogs after return of spontaneous circulation at rest (**A**) and at stress (**B**).

There was a significant segmental myocardial hypoperfusion in the resting state after ROSC, especially in the anterior, lateral, and posterior walls of the left ventricle (Figure 2A). There was no significant increase in MBF even under dobutamine stress. The MBF of the EECP group was significantly higher than that of the Control group, both at rest (Figure 2B) and under dobutamine stress condition (Figure 2C). Multivariate regression analysis showed that MBF was highly correlated with the survival time of dogs. The higher the MBF value, the longer the dog survived (Figure 3A and B).

## Discussion

Our study showed that dogs that had a long-term (12 min) cardiac arrest, followed by severe heart failure, and showed symptoms of peripheral circulatory failure, such as inadequate tissue perfusion, severe acidosis, blood lactic acid accumulation, accompanied by lung damage (increased lung water content and PVPI). Tissue oxygen metabolism was abnormal, manifested by DO_2_ decreases and VO_2_ increases. EECP improved the cardiac function of dogs after ROSC in multiple aspects, such as increasing CPP and MBF, preserving myocardial contractility, reducing peripheral vascular resistance to reduce cardiac afterload, increasing CO to improve tissue perfusion, promoting venous return to increase cardiac preload, and increasing stroke volume. EECP improved blood rheology and helped in improving microcirculatory blood flow in other tissues, including the myocardium.

The EECP is an important research apparatus in our laboratory, similar to the intra-aortic balloon pump (IABP), as it enhances coronary blood flow. However, unlike IABP, a retrograde venous pulse is also generated, which increases venous return and shear stress significantly (16). Cardiac arrest, subsequent to cardiopulmonary resuscitation, and cardiac dysfunction after ROSC impair blood flow and decrease shear stress, which causes endothelial cells to produce NO and ET-1 as well as antithrombotic and anticoagulant agents. Hence, increasing the shear stress during or after ROSC could improve the outcome (22). Previous studies in our laboratory showed that the use of EECP during CPR or after ROSC could improve hemodynamic parameters significantly, thereby improving the ROSC rate and neurologic outcome (14,23–25).

EVLW has shown to have a clear correlation with the severity of acute respiratory distress syndrome, length of ventilation days, intensive care unit stay, and mortality, and it is superior to assessment of lung edema by chest X-ray (26). In the current study, EVLW and PVPI were significantly increased after ROSC. Based on our findings and previous data, we speculate that dogs experienced lung injury after ROSC in addition to heart failure. Lung injury often occurs after CPR, and the main pathological features are pulmonary hemorrhage, pulmonary edema, and atelectasis (27,28). Bacterial infection and inappropriate ventilation parameters, such as tidal volume and high positive end-expiratory pressure, may worsen lung injury after ROSC. Therefore, early detection of lung injury and proper management after ROSC are of great significance for patients. Monitoring PiCCO2 contributes to the early discovery of lung injury after ROSC.

Hemorheology is classified as macroscopic and microscopic. The former includes blood viscosity, plasma viscosity, erythrocyte sedimentation rate, and blood and wall stress distribution, whereas the latter includes erythrocyte aggregation, erythrocyte deformability, platelet aggregation, and platelet adhesion; it is also known as cell rheology (29). Commonly used parameters in clinical practice include blood flow velocity, plasma viscosity, erythrocyte aggregation index, and hematocrit. Abnormalities in these parameters can cause microcirculatory disorders, causing tissue and cellular ischemia and hypoxia. In this study, we are the first to verify that EECP can directly improve the main indicators of hemorheology, such as increased blood flow velocity, decreased plasma viscosity, and erythrocyte aggregation index, and partially affect hematocrit; these changes are beneficial for improving microcirculation.

There are no studies examining the correlation between global hemodynamic and tissue perfusion parameters during cardiac arrest and resuscitation (30,31). A study showed that global hemodynamic parameters do not correlate with oxygenation and tissue perfusion values (18); there may be cardiac dysfunction after ROSC with coronary blood flow not reducing the condition. Thus, it is necessary to monitor the microcirculatory blood flow of the myocardium to evaluate treatment effectiveness. The new PET tracer, ^18^F-flurpiridaz, with high myocardial extraction, allows quantitative MBF estimation from dynamic PET data and tracer kinetic modeling (32). MPI is a simple and non-invasive method for detecting myocardial ischemia, monitoring MBF after ROSC. In this study, EECP increased CPP and MBF values, and MBF values were closely related to the survival time of dogs, which was consistent with the finding that hemodynamic-directed cardiopulmonary resuscitation improves short-term survival reported in previous clinical studies (33,34). Clinically, routine monitoring of CPP is not feasible, and non-invasive MBF monitoring has great advantages.

A clinically useful indication of the findings of this study is that strategies for improving tissue blood flow after ROSC are more helpful in improving the prognosis of patients. There are some limitations in this study. First, we did not evaluate the neurological function of animals after ROSC. This study focused on the effects of EECP on hemodynamics, so the observation time was relatively short. If the observation time could be extended to 72 h or longer, the hemodynamic changes caused by EECP could be reflected fully to benefit neurological function. Second, only the myocardial blood flow at baseline and 4 h after ROSC were observed. Although this can give important clinical indications, continuous observation can reflect the dynamic changes of myocardial blood flow after ROSC.

We conclude that the PiCCO2 monitoring system demonstrated that EECP improved the hemodynamics of dogs in multiple aspects. It increased myocardium blood flow, subsequently improving cardiac function, and ultimately improving survival time of animals after ROSC.

## Supplementary material

Click here to view [pdf].
